# Cardiorespiratory fitness and risk of site‐specific cancers: a long‐term prospective cohort study

**DOI:** 10.1002/cam4.1043

**Published:** 2017-03-20

**Authors:** Trude E. Robsahm, Ragnhild S. Falk, Trond Heir, Leiv Sandvik, Linda Vos, Jan Erikssen, Steinar Tretli

**Affiliations:** ^1^ Department of ResearchCancer Registry of NorwayPB 5313 MajorstuenOslo0304Norway; ^2^ Oslo Centre for Biostatistics and EpidemiologyOslo University HospitalPB 4950 NydalenOslo0424Norway; ^3^ Oslo Ischemia StudyOslo University HospitalOsloNorway; ^4^ Department of Public Health and General PracticeNorwegian University of Science and TechnologyPB 8905Trondheim7491Norway

**Keywords:** Cancer risk, cardiorespiratory fitness, measurement, prevention

## Abstract

Based on self‐reported physical activity, there is epidemiologic evidence for a beneficial relation between physical activity and colon cancer in men, but findings for other cancers are inconclusive. Measured cardiorespiratory fitness (CRF) can provide knowledge about the cancer‐preventive value of physical activity. We aimed to assess relationships between CRF and risk of site‐specific cancers. A cohort of 1997 healthy Norwegian men, aged 40–59 years at inclusion in 1972–1975, was followed for cancer throughout 2012 using data from the Cancer Registry of Norway. CRF was measured by a maximal exercise bicycle test at inclusion. Relationships between CRF and site‐specific cancers were estimated using Cox regression, adjusted for age, body mass index, and smoking. During follow‐up, 898 cancer cases were diagnosed in 758 men. When comparing men in CRF tertile 1 with men in tertiles 2 and 3, respectively, we found decreased risk of proximal colon cancer in tertile 2 (HR: 0.30, 95% CI: 0.13–0.73) and decreased risk of cancers of lung (0.39 95% CI: 0.22–0.66), pancreas (0.32 95% CI: 0.10–1.00), and bladder (HR: 0.40 95% CI: 0.21–0.74) in tertile 3. Furthermore, a significant trend for lower risk by increasing CRF tertile was found for cancers of proximal colon, lung, and bladder (*P*‐value for trend <0.05). For other cancer sites, no significant association was found. Our results indicate that high midlife CRF may have cancer‐preventive value.

## Introduction

Cancers are among the leading causes of morbidity and mortality worldwide, with approximately 14 million new cases and 8.2 million cancer‐related deaths in 2012 1. The number of new cases is expected to rise by about 70% over the next two decades 1 and prevention of cancers is an important health issue.

Physical activity influences several biologic mechanisms (hormonal, immunologic, and mechanical), and may reduce the risk of several cancers 2, 3, 4, 5. In a large number of epidemiologic studies, the relationship between physical activity and cancer risk has been investigated. However, evidence of a beneficial effect in men is only found for colon cancer 6, 7. The unclarified relationship between physical activity and cancer development may be due to difficulties in obtaining reliable data on physical activity habits. Self‐reported physical activity, typically used in epidemiological research, may underestimate the association between physical activity and health outcomes 8, 9.

Physical fitness, a set of physiologic attributes that are enhanced through regular physical activity, is less prone to misclassification and may better capture health consequences of an active versus sedentary lifestyle than self‐reported activity. Most common in use in health studies is the measurement of cardiorespiratory fitness (CRF), which reflects the ability of the body's circulatory and respiratory systems to supply oxygen during sustained physical activity. CRF thus constitutes an objective measure of aerobic activity performed over time 10, although it cannot capture all physical activity (e.g., strength, mobility, etc.). Using gold‐standard measures, the correlation between CRF and physical activity is reported to be modest, but with regard to cardio‐protective effects, CRF was found to be more efficient than physical activity 11. This provides an expectation of a stronger association between CRF and cancer than found for self‐reported physical activity and it may add knowledge about the preventive value of physical activity level.

Several studies have reported an inverse relationship between CRF and cancer mortality in men 12, 13, 14, 15, 16, 17, 18, 19, 20, 21, 22, whereas with regard to cancer risk, very few studies are based on measured CRF 19, 23, 24, 25. The results, however, indicate an inverse association between CRF and risk of total cancer 19, 24, 26, lung cancer 19, 24, colon cancer 24, and cancers of the gastrointestinal tract 19, while for prostate cancer, the results are inconclusive 19, 23, 24, 25. These findings provide further support and more studies are requested to reveal the level of CRF necessary to influence risk of site‐specific cancer 24. There is also a need to examine whether CRF is related to risk of cancer at other sites, than those previously focused on.

Based on a male cohort of 1997 initially healthy middle‐aged men, we aimed to assess relationships between CRF levels and risk of site‐specific cancers.

## Material and Methods

The study is based on the Oslo Ischemia Cohort 27, linked to data from the Cancer Registry of Norway.

### Data sources

The Oslo Ischemia Study is a comprehensive health survey established in 1972, aimed to examine the prevalence and development of coronary heart disease and other cardiovascular diseases in a healthy male population 27. In total, 2341 employed men in the age‐group 40–59 years were invited, of whom 2014 (86%) apparently healthy men participated by completing the study protocol. Men were defined as healthy as determined by a thorough screening of health records and by medical examinations. Inclusion required absence of Cardiovascular Diseases (CVD), diabetes, cancer or other advanced disease (pulmonary, renal, and liver), or disorders that made it impossible to undertake a symptom‐limited exercise test. Men who regularly were taking medications that might affect the exercise test or heart rate response were excluded. Details about the selection criteria are presented elsewhere 27, 28, 29. At baseline, after 12 h of fasting and 8 h nonsmoking, a comprehensive clinical examination was conducted, including measurements of height, weight, blood pressure, and a panel of blood tests 27. CRF was assessed by an incremental bicycle exercise test, with a duration of 6 min on each step, starting at 100 W. The load was incremented by 50 W per step until volitional exhaustion 27. Based on this cohort, two studies have reported results on cancer—one studying the relation between cholesterol and prostate cancer^30^, another investigating associations between CRF and overall cancer risk and death, and correlations between CRF and self‐reported physical activity 31.

The Cancer Registry of Norway has, since the establishment in 1953, registered data on all malignancies diagnosed in the total Norwegian population. Mandatory reporting from several independent sources ensures completeness and high data quality 32. The Oslo Ischemia study was linked to the Cancer Registry and the Cause of Death Registry, using the unique 11‐digit personal identification number, assigned to all Norwegians in 1960 and thereafter to all newborns and persons residing in Norway. The linkage gave information on cancer and vital status. Permission to link the data was provided by the Regional Committees for Medical and Health Research Ethics.

### Exposure variables

Cardiovascular fitness was measured as total work (sum of work performed in the bicycle test) divided by body weight, kJ/kg, in tertiles, giving the tertile limits: 1: <118.9 (mean 91.9); 2: 119–161.4 (mean 139.1); 3: >161.5 (mean 207.9). Age at inclusion was divided into four groups (<45, 45–49, 50–54, 55 + years). Individual body mass index (BMI) was calculated based on the objective measurements of height and weight (body weight/height^2^, kg/m^2^), divided into two categories: low/normal weight (BMI < 25) and overweight/obese (BMI ≥ 25). Based on self‐reported information on smoking, the men were categorized as ever and never smokers.

Of the 2014 men, two were excluded due to missing vital status data and 15 were excluded due to a cancer diagnosis prior to date of the first examination, leaving 1997 men for analyses.

Information on diet and alcohol consumption, which are factors associated with risk of cancers in men, in particular of the gastrointestinal tract 6, 7_,_ was not available. Nor were we able to consider the role of socioeconomic factors.

### Statistical analyses

The study cohort was followed from the date of health examination (inclusion) to the date of cancer diagnosis, date of death, emigration, or end of follow‐up, at December 31, 2012, whichever occurred first. Descriptive analyses were conducted for baseline characteristics, presented as means (with SD, standard deviations) and percentages. Cancer cases were categorized into cancer sites, using ICD‐10 codes for each cancer diagnosis.

Cox regression models were conducted to evaluate the relationship between CRF and risk of specific cancers. Time to event was calculated as time from date of inclusion to date of primary diagnosis for each cancer‐site. Effect estimates are presented as hazard ratios (HRs) with 95% confidence intervals (CI). Potential confounding factors included in the final Cox model were age, BMI, and smoking. In each model we verified that the proportional‐hazards assumption was met based on analyzing Schoenfeld residuals. *P*‐values for trend were estimated by increasing CRF tertile, for each cancer site.

The relationship between CRF and cancer was assessed for cancer sites that have been of main focus in relation to physical activity 6: colon, rectum, lung, pancreas, kidney, bladder, prostate, skin, lymphoma and leukemia, and cancers of the central nervous system. In addition, we assessed the relationship for mouth/pharynx, esophagus/stomach, liver/gallbladder/bile duct, as suggested cancer prevention mechanisms of physical activity also may apply for these cancer sites.

Colonic subsites are suggested to be differently associated with lifestyle variables such as physical activity, smoking, alcohol, hormones, and BMI 31; therefore, separate analyses were conducted for cancer risk of the proximal and distal parts of the colon. For prostate cancer, the analyses were stratified by stage of disease, due to potential different diagnostic intensities of prostate cancer in physically active and inactive men 3. For skin cancer, separate analyses were conducted by anatomic site, to allow taking excess exposure from ultraviolet radiation (UVR) into account 33.

Finally, a corresponding Cox model was used for sensitivity analyses. To eliminate the possibility that a low score CRF resulted from an ongoing cancer disease (reverse causality), this analysis was restricted to men still alive and cancer‐free 10 years after baseline.

All statistical analyses were performed using Stata 14 34. The statistical significance level was set at 5%.

## Results

Table 1 shows the characteristics of the study cohort at baseline. For all men together, mean age at baseline was 49.8 years (SD: 5.5). With increasing age category, the percentage of men in the lowest fit tertile increased, while the percentage of men in the highest fit tertile decreased. For those youngest at baseline (<45 years), the majority were in CRF tertile 3 (57.8%), while for the oldest (≥55 years), the majority were in CRF tertile 1 (59.7%). Mean BMI was similar by CRF tertiles, but a larger proportion of the men with BMI <25 were in CRF tertile 3 (38.4%) than among men with BMI ≥25 (25%). Nearly 75% of the men were ever‐smokers, with the lowest proportion in the third CRF tertile (69.9%). Mean cholesterol and blood pressure were within normal range for men in the age group, with lower means by higher levels of CRF.

**Table 1:** Study population characteristics at the health examination at baseline, according to cardiorespiratory fitness (CRF) tertiles (*n* = 1997)

Characteristics	CRF tertiles [kJ/kg]		
	1 [<118.9] *n* = 667	2 [119‐161.4] *n* = 665	3 [≥161.5] *n* = 665
Age years, mean (SD)	52.4 (5.2)	49.9 (5.0)	46.9 (4.8)
<45, *n* (%)	66 (14.1)	132 (28.1)	271 (57.8)
45–49, *n* (%)	139 (24.5)	206 (36.3)	223 (39.2)
50–54, *n* (%)	218 (39.6)	209 (38.0)	123 (22.4)
≥55, *n* (%)	245 (59.7)	118 (28.8)	47 (11.5)
CRF kJ/kg, mean (SD)	91.9 (19.9)	139.1 (12.3)	207.9 (50.7)
Weight kg, mean (SD)	76.8 (11.1)	77.2 (9.5)	76.5 (9.0)
Height cm, mean (SD)	175.3 (6.1)	176.8 (5.9)	178.1 (6.2)
BMI kg/m^2^, mean (SD)	24.9 (3.1)	24.7 (2.7)	24.1 (2.3)
<25, *n* (%)	365 (30.0)	386 (31.7)	467 (38.3)
≥25, *n* (%)	312 (39.6)	279 (35.4)	197 (25.0)
Smoking ever, *n* (%)	521 (78.1)	509 (76.5)	465 (69.9)
Cholesterol mmol/L, mean (SD)	6.8 (1.2)	6.7 (1.1)	6.5 (1.2)
Blood pressure, mm/Hg, mean (SD)			
Systolic	135.3 (19.8)	129.9 (16.8)	125.1 (15.0)
Diastolic	89.3 (10.8)	87.1 (10.0)	84.9 (9.8)

SD, standard deviation; BMI, body mass index.

Mean follow‐up time was 26.2 years (SD: 10.3, range: 0.07–40.3). In total, 898 cancer cases were diagnosed in 758 men, where 84% had one diagnosis, 14% had two diagnoses, and 2.5% had three or several diagnoses. Table 2 presents the number of cancer diagnoses by site, illustrating that prostate was the most frequent site, followed by lung and colon. The table also shows the number of site‐specific cancer diagnoses according to CRF tertiles. When comparing the cancer occurrence in the study cohort with the general male population in the area the men were recruited from, no major differences were revealed 30.

**Table 2:** Numbers of the first primary cancers by site diagnosed from baseline (date of health examination) to the end of follow‐up (2012), according to tertiles of cardiorespiratory fitness (CRF)

Cancer site	ICD‐10	Total	CRF tertiles		
			1	2	3
All sites	C00‐96	898	278	313	307
Mouth, pharynx	C00‐14	24	10	8	6
Esophagus, stomach	C15, C16	43	13	14	16
Colon	C18	87	35	23	29
Proximal	C18.0‐18.5	46	21	7	18
Distal	C18.6‐18.7	41	14	16	11
Rectum, anus	C19‐21	44	10	19	15
Liver, gallbladder/bile ducts	C22, C23‐24	13	4	6	3
Pancreas	C25	29	14	11	4
Lung, trachea	C33‐34	109	46	41	22
Prostate	C61	213	52	71	90
Kidney, renal pelvis	C64, C65	34	11	12	11
Bladder, ureter, urethra	C66‐68	76	32	26	18
Skin, melanoma	C43	49	9	15	25
Skin, nonmelanoma	C44	47	12	14	21
Lymphoma	C81‐90	30	5	13	12
Leukemia	C91‐95	37	9	16	12
Central nervous system	C70‐72/D42‐43	19	5	6	8
Unspecified	C39, C76, C80	23	8	9	6
Other sites^a^		21	3	9	9

a Nose (C30‐31), Larynx (C32), Other digestive organs (C26), Other male genitals (C60, C63), Eye (C69), Endocrine glands (C37/C73‐75), Soft tissue (C48‐49).

Results from the Cox analyses are presented in Table 3, showing the HR for cancer risk by site (with 95% CIs) for CRF tertiles 2 and 3 relative to tertile 1, adjusted for age, BMI, and smoking. For colon, men in the second and third CRF tertile had noticeably lower HRs compared to men in tertile 1, although the differences were not statistically significant. When separating colonic subsites, the number of cases was near equally distributed at the proximal and distal part. For the proximal colon, a significantly reduced cancer risk was found for men in the second CRF tertile, compared to tertile 1, while no significant relationship was found between CRF and cancer of the distal colon and neither for cancer of the rectum (Table 3). Relative to CRF tertile 1, men in CRF tertile 3 had significantly lower risk of lung cancer, pancreatic cancer, and bladder cancer. Furthermore, a significant trend for lower risk by increasing CRF tertile was found for cancers of proximal colon, lung, and bladder (*P*‐value for trend <0.05).

**Table 3:** Hazard ratio (HR) and 95% confidence intervals (CIs) for site‐specific cancer by tertiles of cardiorespiratory fitness (CRF), adjusted for age, body mass index, and smoking at baseline, and *P*‐values from trend‐tests

	CRF tertiles [kJ/kg, tertile limits, mean per tertile]			Trend
	1 [<118, 91.9]	2 [119–161, 139.1]	3 [>161, 207.9]	
Cancer site	HR	HR (95% CI)	HR (95% CI)	*P*‐value
Mouth/pharynx	1.00	0.64 (0.25,1.65)	0.41 (0.14,1.19)	0.25
Esophagus/stomach	1.00	1.09 (0.50,2.36)	1.24 (0.56,2.77)	0.86
Colon	1.00	0.60 (0.35,1.01)	0.69 (0.40,1.17)	0.15
Proximal	1.00	0.30 (0.13,0.73)	0.69 (0.34,1.39)	0.03
Distal	1.00	1.00 (0.48,2.08)	0.65 (0.28,1.50)	0.49
Rectum, anus	1.00	1.65 (0.76,3.61)	1.16 (0.49,2.73)	0.38
Liver/gallbladder/bile duct	1.00	1.62 (0.44,5.97)	0.81 (0.16,4.01)	0.57
Pancreas	1.00	0.80 (0.35,1.80)	0.32 (0.10,1.00)	0.15
Lung, trachea	1.00	0.78 (0.50,1.19)	0.39 (0.23,0.66)	0.002
Prostate	1.00	1.17 (0.81,1.69)	1.20 (0.83,1.74)	0.59
Kidney, renal pelvis	1.00	0.86 (0.37,1.99)	0.65 (0.27,1.58)	0.63
Bladder, ureter, urethra	1.00	0.69 (0.41,1.18)	0.40 (0.21,0.74)	0.01
Skin cancer	1.00	1.31 (0.73,2.35)	1.70 (0.96,3.00)	0.18
Melanoma	1.00	1.48 (0.63,3.42)	2.19 (0.99,4.96)	0.14
Nonmelanoma	1.00	1.06 (0.48,2.35)	1.20 (0.55,2.60)	0.88
Lymphoma	1.00	2.44 (0.85,6.99)	1.89 (0.62,5.78)	0.25
Leukemia	1.00	1.86 (0.80,4.30)	1.26 (0.49,3.21)	0.30
Central nervous system	1.00	1.03 (0.31,3.44)	1.27 (0.38,4.18)	0.90
Unspecified	1.00	0.97 (0.37,2.58)	0.59 (0.19,1.82)	0.59
Other sites^a^	1.00	3.16 (0.83,11.94)	3.29 (0.81,13.31)	0.19

a Nose (C30‐31), Larynx (C32), Other digestive organs (C26), Other male genitals (C60, C63), Eye (C69), Endocrine glands (C37/C73‐75), Soft tissue (C48‐49).

No significant relation was found between CRF and kidney cancer (Table 3). Neither was the risk of prostate cancer related to CRF nor in analyses stratified by stage at diagnosis (results not shown).

For melanoma skin cancer, the association tends to go in the opposite direction, although not statistically significant. Subanalyses by anatomic site of the melanoma revealed that the risk increase was valid for head and trunk only (results not shown).

For cancer located at mouth/pharynx, the relation tended to be inverse, although not statistically significant (Table 3). For other cancer sites, no significant relations with CRF were found (Table 3).

## Discussion

Based on the present cohort, of initially healthy middle‐aged men, we previously have found that high CRF was associated with lower overall cancer risk, cancer mortality, and cancer case fatality, compared to men with low CRF 26. In this study, we found that high midlife CRF was associated with decreased risk of cancers of lung, pancreas, and bladder, and that medium CRF was associated with decreased risk of proximal colon cancer, when compared to low CRF. For other cancer sites, no significant associations were found.

A recently published pooled analysis, based on 1.4 million participants and about 180 000 cancer cases during 11 years of follow‐up 35, reports that higher levels of self‐reported leisure time activity were associated with lower risk of nine cancer sites in men. In this study, based on CRF measurement, we found the same relationship for several cancer sites, with an even larger magnitude, although the number of cancer cases was small.

Colon cancer has received much research attention with respect to the role of physical activity, and is the only site with sufficient evidence for a beneficial role of physical activity in men 6, 7. In general, the risk reduction reported is approximately 20‐25% 3. In this study, based on CRF measurements, men with the lowest CRF seem to have the highest risk of colon cancer, compared to men having higher CRF, but the difference was not statistically significant. To our knowledge, only one study had previously examined the relationship between CRF and colon cancer risk, and the results show an inverse graded relationship 24. This study was conducted with a similar design and methods as we included men only and in corresponding age groups. An important difference, however, is that it was a substantially larger study, including 181 colon cancers 24. Moreover, biological differences between colonic segments 36, 37 have raised questions on whether lifestyle factors affect the cancer risk of colon subsites differently. Nevertheless, two meta‐analyses report that physical activity was inversely associated with cancer of the proximal and distal colon, with the same magnitude for both subsites 31, 38. To our knowledge, no studies have examined the relation between CRF and site‐specific colon cancer. Our analyses revealed decreased risk of proximal colon cancer for men in CRF tertile 2, compared to men in tertile 1, and the *P*‐value for trend was 0.03. For distal colon, no significant association was found. Our findings may indicate a subsite difference between the colonic segments, although it has to be kept in mind that we were not able to take alcohol consumption or dietary factors into account.

There is no epidemiologic evidence for a relationship between physical activity and rectum cancer 6, 7. Nor did we, in this study, observe any relation between CRF and rectum cancer. To our knowledge, this is the first study based on CRF that reports a site‐specific result for rectum. The study by Laukkanen et al. 19, however, found an inverse association between CRF and all gastrointestinal tract cancers (*n* = 92), including the rectum, but the number of rectum cancers was not given.

For lung cancer, we found an evident inverse relationship between CRF and risk. Compared to men in CRF tertile 1, men in tertile 3 had a 61% reduced risk, when age, BMI, and smoking were taken into account. The magnitude of the risk reduction is larger than reported from studies based on self‐reported physical activity (20–40%) 3, 35, but was in line with results reported from the two present studies based on CRF 19, 24. These studies were conducted with similar design and methods as this study, including men in corresponding age groups. Together, these three studies indicate that a high CRF may prevent the development of lung cancer. Although the main risk factor for this disease (smoking) has been adjusted for, we cannot exclude the possibility of residual confounding. The main mechanism of physical activity suggested to explain this relation is improved pulmonary function that shortens exposure time and reduced concentration of carcinogenic agents in the lungs 3. Another mechanism resulting from aerobe exercise, which has been observed in mice, is an increase of immune cells and generation of a cancer‐inhibiting environment in the tissue 39.

In this study, a high CRF was associated with 68% and 60% reduction in risk of pancreatic and bladder cancer, respectively. To our knowledge, no studies have reported relations between CRF and these cancer sites. WHO/NCI has classified the potential inverse relation between physical activity and pancreatic cancer as “limited‐suggestive” 6, 7, while for bladder cancer no conclusion has been drawn 6, 7. The recently published pooled analysis of 12 cohort studies reported that physically active men had a slightly reduced risk of bladder cancer, but no relation was found for pancreatic cancer 35. For these cancers, few carcinogenic agents have been given with sufficient evidence, except for tobacco smoking 40. In the present cohort, a large proportion of the participants reported to be ever smokers (74.9%), with a slightly decreasing proportion by increasing CRF tertile. Although smoking was adjusted for in the analyses, this did not affect the association between CRF and risk of these cancers. For bladder cancer, occupational exposure (e.g., aluminum industry) is also assumed to increase the risk 40. The cohort in this study was recruited from companies without that kind of exposure and, thus, any impact of such agents on our results is unlikely. Biologic mechanisms of physical activity may explain the relationship between CRF and cancers of the pancreas and the bladder. Physical activity prevents insulin resistance and reduces gut transit time, which may have beneficial effect on bile secretion and pancreatic activity 41. Further, physical activity prevents obesity, which may have a positive impact on the risk of bladder cancer 42.

A large number of studies have investigated the relationship between self‐reported physical activity and prostate cancer, showing inconsistent results 3, 6. Further, in the recently published pooled analysis, men who reported the highest physically active level were found to have a slightly elevated risk of prostate cancer 35. No studies based on measured CRF have been able to clarify this relation. To our knowledge, five studies report risk estimates for the relation between CRF and risk of prostate cancer—two report no association 19, 24, two report a positive association 25, 30, while one reports an inverse association for men aged <60 years 23. In this study, no significant relation between CRF and prostate cancer was found. An issue often discussed in studies on prostate cancer is the possible selection bias of higher diagnostic intensity of small tumors in physically active men, as they may belong to a higher socioeconomic level and are more concerned about health. In this study, information on socioeconomic variables (i.e., education, income) was not available. An approach to overcome this limitation, sensitive analyses by stage of disease, was conducted, expecting a positive association for local stage disease at diagnosis, but not for advanced stage disease. This analysis revealed no significant findings and no differences between the stage groups.

For melanoma skin cancer, we observed elevated HR for men in CRF tertile 3, compared to those in tertile 1, indicating a positive relationship. Also, studies based on self‐reported physical activity report a positive relation to skin cancer 35. Physically active people may spend more time outdoor than inactive individuals and may therefore have higher exposure to UVR from the sun, the main risk factor for skin cancer 40. Additional analyses, stratified by anatomic site, showed that the elevated risk was restricted to tumors located at the head and the trunk, giving support for this explanation.

Studies examining relations between physical activity and lympho‐hematopoietic cancers indicate an inverse association for non‐Hodgkin's lymphoma and leukemia 3, 35, 43. To our knowledge, this study is the first that investigated the association between CRF and these cancers, revealing no significant associations. Prevention of obesity is one mechanism of physical activity suggested to explain the inverse relationship to hematologic cancers 43. BMI, measured at baseline, was taken into account in our analyses, but did not significantly influence the association between CRF and cancer risk. However, we cannot exclude a possible impact of weight gain after baseline. Another beneficial effect of physical activity suggested to play a role is improved immune function 39, 43, 44. On the other hand, regularly intense exercise is related to immune suppression 45, 46 and increased susceptibility to infections associated with lymphoid cancers 47. Unfortunately, information on immunologic factors or prevalence of infections was not available and, thus, not taken into account in our analyses.

For other cancer forms, no significant relation to CRF was found, but of interest might be the lower HRs of mouth and pharynx cancers in CRF tertiles 2 and 3, compared to tertile 1, although not statistically significant. In general, physical activity has not been related to cancer at these sites 6. The main risk factors for mouth and pharynx cancers are tobacco, alcohol, and human papillomavirus (HPV) 40. In this study, we adjusted for smoking, but were not able to adjust for alcohol intake and HPV infections, as we had no information about these factors.

The study has several strengths. Primarily this entails complete information on CRF in 1997 men**,** who were prospectively followed up for cancer outcomes over a 40‐year period. The midlife CRF measurements is a reliable measure of aerobic activity over time, which is a key factor that has to be emphasized, although we are aware that a CRF test not capture all kind of activity (i.e., light activity that does not impact the aerobic capacity). From the Cancer Registry of Norway we have complete and valid information on cancer diagnoses and cause of death during the time‐span covered, and we have individual‐level information on several potential confounding variables. Lastly, the cohort is shown to be representative of this age group of men, with regard to cancer occurrence in the counties were recruited from (Oslo and Akershus), within the given time‐period 30. On the other hand, the study has limitations that need to be considered when interpreting the results. First, the study has a relatively small sample size, resulting in few cases of each cancer site and thus low power in cancer‐specific analyses. Second, all variables were assessed at baseline, which make us unable to account for changes over the life course, that may have influenced the observed associations and, with regard to the smoking variable, lack of information on pack‐years is a weakness. Third, although one main criterion for cohort inclusion was good health, a low‐score CRF test may result from an ongoing undiagnosed disease. To overcome this, we conducted analyses excluding the first 10 years after enrollment. This analysis, however, gave similar results and thus rejects such an explanation. Fourth, we lack information on dietary factors and alcohol intake, factors that are associated with cancers of the gastrointestinal tract. Nor were we able to consider socioeconomic factors (i.e., education, income), which is a limitation as health concerns vary between socioeconomic levels.

## Conclusion

In men, a beneficial relation between physical activity and colon cancer is established, but for other cancers, the relationship is inconclusive. Little is known about the relations between CRF and cancer risks. This 40‐year follow‐up study of initially healthy middle‐aged men reveals a potentially adverse effect of having low CRF, for risk of several cancers. Compared to men with low CRF, men with higher levels have reduced risk of cancers in the proximal colon, lung, pancreas, and bladder. Despite the fact that the study is small, our results are in line with previous findings, which strengthen the role of CRF as a potential cancer preventive factor.

## Conflict of Interest

None declared.

## References

[1] Stewart, B. W. , and C. P. Wild . 2014 World Cancer Report 2014 Lyon. International Agency for Research on Cancer, France. 39432694

[2] Friedenreich, C. M. 2001 Physical activity and cancer prevention: from observational to intervention research. Cancer Epidemiol. Biomarkers Prev. 10:287–301. 11319168

[3] Friedenreich, C. M. , H. K. Neilson , and B. M. Lynch . 2010 State of the epidemiological evidence on physical activity and cancer prevention. Eur. J. Cancer 46:2593–2604. 20843488 10.1016/j.ejca.2010.07.028

[4] Friedenreich, C. M. , and M. R. Orenstein . 2002 Physical activity and cancer prevention: etiologic evidence and biological mechanisms. J. Nutr. 132:3456S–3464S. 12421870 10.1093/jn/132.11.3456S

[5] Pedersen, L. , J. F. Christensen , and P. Hojman . 2015 Effects of exercise on tumor physiology and metabolism. Cancer J. 21:111–116. 25815851 10.1097/PPO.0000000000000096

[6] World Cancer Research Fund/American Institute for Cancer Research, Food, Nutrition, Physical Activity, and the Prevention of Cancer. A Global Perspective. AICR, 2007.

[7] World Cancer Reserach Fund/American Institute for Cancer Research, Continuous Update Project report. Food, Nutrition, Physical Activity, and the Prevention of Colorectal Cancer, 2011.

[8] Steene‐Johannessen, J. , S. A. Anderssen , H. P. van der Ploeg , I. J. Hendriksen , A. E. Donnelly , S. Brage , et al. 2016 Are Self‐report Measures Able to Define Individuals as Physically Active or Inactive? Med. Sci. Sports Exerc. 48:235–244. 26322556 10.1249/MSS.0000000000000760 PMC6235100

[9] LaMonte, M. J. , and S. N. Blair . 2006 Physical activity, cardiorespiratory fitness, and adiposity: contributions to disease risk. Curr. Opin. Clin. Nutr. Metab. Care 9:540–546. 16912548 10.1097/01.mco.0000241662.92642.08

[10] Åstrand, P.‐O . 2003 Textbook of work physiology: physiological bases of exercise. Human Kinetics Publishers, United Kingdom.

[11] Dvorak, R. V. , A. Tchernof , R. D. Starling , P. A. Ades , L. DiPietro , and E. T. Poehlman . 2000 Respiratory fitness, free living physical activity, and cardiovascular disease risk in older individuals: a doubly labeled water study. J. Clin. Endocrinol. Metab. 85:957–963. 10720023 10.1210/jcem.85.3.6432

[12] Zhang, P. , X. Sui , G. A. Hand , J. R. Hebert , and S. N. Blair . 2014 Association of changes in fitness and body composition with cancer mortality in men. Med. Sci. Sports Exerc. 46:1366–1374. 24276414 10.1249/MSS.0000000000000225 PMC4031307

[13] Sui, X. , D. C. Lee , C. E. Matthews , S. A. Adams , J. R. Hebert , T. S. Church , et al. 2010 Influence of cardiorespiratory fitness on lung cancer mortality. Med. Sci. Sports Exerc. 42:872–878. 19996990 10.1249/MSS.0b013e3181c47b65 PMC2859116

[14] Schmid, D. , and M. F. Leitzmann . 2015 Cardiorespiratory fitness as predictor of cancer mortality: a systematic review and meta‐analysis. Ann. Oncol. 26:272–278. 25009011 10.1093/annonc/mdu250

[15] Sawada, S. S. , I. M. Lee , H. Naito , R. Kakigi , S. Goto , M. Kanazawa , et al. 2014 Cardiorespiratory fitness, body mass index, and cancer mortality: a cohort study of Japanese men. BMC Public Health 14:1012. 25261876 10.1186/1471-2458-14-1012 PMC4190338

[16] Peel, J. B. , X. Sui , C. E. Matthews , S. A. Adams , J. R. Hebert , J. W. Hardin , et al. 2009 Cardiorespiratory fitness and digestive cancer mortality: findings from the aerobics center longitudinal study. Cancer Epidemiol. Biomarkers Prev. 18:1111–1117. 19293313 10.1158/1055-9965.EPI-08-0846 PMC2688961

[17] Lee, C. D. , X. Sui , S. P. Hooker , J. R. Hebert , and S. N. Blair . 2011 Combined impact of lifestyle factors on cancer mortality in men. Ann. Epidemiol. 21:749–754. 21683616 10.1016/j.annepidem.2011.04.010 PMC3166420

[18] Lee, C. D. , and S. N. Blair . 2002 Cardiorespiratory fitness and smoking‐related and total cancer mortality in men. Med. Sci. Sports Exerc. 34:735–739. 11984287 10.1097/00005768-200205000-00001

[19] Laukkanen, J. A. , E. Pukkala , R. Rauramaa , T. H. Makikallio , A. T. Toriola , and S. Kurl . 2010 Cardiorespiratory fitness, lifestyle factors and cancer risk and mortality in Finnish men. Eur. J. Cancer 46:355–363. 19683431 10.1016/j.ejca.2009.07.013

[20] Kampert, J. B. , S. N. Blair , C. E. Barlow , and H. W. 3rd Kohl . 1996 Physical activity, physical fitness, and all‐cause and cancer mortality: a prospective study of men and women. Ann. Epidemiol. 6:452–457. 8915477 10.1016/s1047-2797(96)00059-2

[21] Farrell, S. W. , G. M. Cortese , M. J. LaMonte , and S. N. Blair . 2007 Cardiorespiratory fitness, different measures of adiposity, and cancer mortality in men. Obesity (Silver Spring) 15:3140–3149. 18198325 10.1038/oby.2007.374

[22] Evenson, K. R. , J. Stevens , J. Cai , R. Thomas , and O. Thomas . 2003 The effect of cardiorespiratory fitness and obesity on cancer mortality in women and men. Med. Sci. Sports Exerc. 35:270–277. 12569216 10.1249/01.MSS.0000053511.02356.72

[23] Oliveria, S. A. , H. W. 3rd Kohl , D. Trichopoulos , and S. N. Blair . 1996 The association between cardiorespiratory fitness and prostate cancer. Med. Sci. Sports Exerc. 28:97–104. 8775361 10.1097/00005768-199601000-00020

[24] Lakoski, S. G. , B. L. Willis , C. E. Barlow , D. Leonard , A. Gao , N. B. Radford , et al. 2015 Midlife cardiorespiratory fitness, incident cancer, and survival after cancer in men: the cooper center longitudinal study. JAMA Oncol. 1:231–237. 26181028 10.1001/jamaoncol.2015.0226 PMC5635343

[25] Byun, W. , X. Sui , J. R. Hebert , T. S. Church , I. M. Lee , C. E. Matthews , et al. 2011 Cardiorespiratory fitness and risk of prostate cancer: findings from the Aerobics Center Longitudinal Study. Cancer Epidemiol. 35:59–65. 20708996 10.1016/j.canep.2010.07.013 PMC3062068

[26] Robsahm, T. E. , R. S. Falk , T. Heir , L. Sandvik , L. Vos , J. E. Erikssen , et al. 2016 Measured cardiorespiratory fitness and self‐reported physical activity: associations with cancer risk and death in a long‐term prospective cohort study. Cancer Med. 5:2136–2144. 27227704 10.1002/cam4.773 PMC4884631

[27] Erikssen, J. , I. Enge , K. Forfang , and O. Storstein . 1976 False positive diagnostic tests and coronary angiographic findings in 105 presumably healthy males. Circulation 54:371–376. 947568 10.1161/01.cir.54.3.371

[28] Sandvik, L. , J. Erikssen , E. Thaulow , G. Erikssen , R. Mundal , and K. Rodahl . 1993 Physical fitness as a predictor of mortality among healthy, middle‐aged Norwegian men. N. Engl. J. Med. 328:533–537. 8426620 10.1056/NEJM199302253280803

[29] Heir, T. , J. Erikssen , and L. Sandvik . 2011 Overweight as predictor of long‐term mortality among healthy, middle‐aged men: a prospective cohort study. Prev. Med. 52:223–226. 21277889 10.1016/j.ypmed.2011.01.010

[30] Heir, T. , R. S. Falk , T. E. Robsahm , L. Sandvik , J. Erikssen , and S. Tretli . 2016 Cholesterol and prostate cancer risk: a long‐term prospective cohort study. BMC Cancer 16:643. 27535659 10.1186/s12885-016-2691-5 PMC4989293

[31] Robsahm, T. E. , B. Aagnes , A. Hjartaker , H. Langseth , F. I. Bray , and I. K. Larsen . 2013 Body mass index, physical activity, and colorectal cancer by anatomical subsites: a systematic review and meta‐analysis of cohort studies. Eur. J. Cancer Prev. 6:492–505. 10.1097/CEJ.0b013e328360f434 23591454

[32] Larsen, I. K. , M. Smastuen , T. B. Johannesen , F. Langmark , D. M. Parkin , F. Bray , et al. 2009 Data quality at the Cancer Registry of Norway: an overview of comparability, completeness, validity and timeliness. Eur. J. Cancer 45:1218–1231. 19091545 10.1016/j.ejca.2008.10.037

[33] Jardine, A. , M. Bright , L. Knight , H. Perina , P. Vardon , and C. Harper . 2012 Does physical activity increase the risk of unsafe sun exposure? Health Promot. J. Austr. 23:52–57. 22730941 10.1071/he12052

[34] StataCorp . 2015 Stata Statistical Software: Release 14 College Station. StataCorp LP, TX.

[35] Moore, S. C. , I. M. Lee , E. Weiderpass , P. T. Campbell , J. N. Sampson , C. M. Kitahara , et al. 2016 Association of leisure‐time physical activity with risk of 26 types of cancer in 1.44 million adults. JAMA Intern. Med. 176:816–825. 27183032 10.1001/jamainternmed.2016.1548 PMC5812009

[36] Li, F. Y. , and M. D. Lai . 2009 Colorectal cancer, one entity or three. J. Zhejiang Univ. Sci. B 10:219–229. 19283877 10.1631/jzus.B0820273 PMC2650032

[37] Iacopetta, B. 2002 Are there two sides to colorectal cancer? Int. J. Cancer 101:403–408. 12216066 10.1002/ijc.10635

[38] Boyle, T. , T. Keegel , F. Bull , J. Heyworth , and L. Fritschi . 2012 Physical activity and risks of proximal and distal colon cancers: a systematic review and meta‐analysis. J. Natl Cancer Inst. 104:1548–1561. 22914790 10.1093/jnci/djs354

[39] Pedersen, L. , M. Idorn , G. H. Olofsson , B. Lauenborg , I. Nookaew , R. H. Hansen , et al. 2016 Voluntary running suppresses tumor growth through epinephrine‐ and IL‐6‐dependent NK cell mobilization and redistribution. Cell Metab. 23:554–562. 26895752 10.1016/j.cmet.2016.01.011

[40] Cogliano, V. J. , R. Baan , K. Straif , Y. Grosse , B. Lauby‐Secretan , F. El Ghissassi , et al. 2011 Preventable exposures associated with human cancers. J. Natl Cancer Inst. 103:1827–1839. 22158127 10.1093/jnci/djr483 PMC3243677

[41] Berrington de Gonzalez, A. , E. A. Spencer , H. B. Bueno‐de‐Mesquita , A. Roddam , R. Stolzenberg‐Solomon , J. Halkjaer , et al. 2006 Anthropometry, physical activity, and the risk of pancreatic cancer in the European prospective investigation into cancer and nutrition. Cancer Epidemiol. Biomarkers Prev. 15:879–885. 16702364 10.1158/1055-9965.EPI-05-0800

[42] Koebnick, C. , D. Michaud , S. C. Moore , Y. Park , A. Hollenbeck , R. Ballard‐Barbash , et al. 2008 Body mass index, physical activity, and bladder cancer in a large prospective study. Cancer Epidemiol. Biomarkers Prev. 17:1214–1221. 18483344 10.1158/1055-9965.EPI-08-0026

[43] Jochem, C. , M. F. Leitzmann , M. Keimling , D. Schmid , and G. Behrens . 2014 Physical activity in relation to risk of hematologic cancers: a systematic review and meta‐analysis. Cancer Epidemiol. Biomarkers Prev. 23:833–846. 24633143 10.1158/1055-9965.EPI-13-0699

[44] Woods, J. A. , V. J. Vieira , and K. T. Keylock . 2009 Exercise, inflammation, and innate immunity. Immunol. Allergy Clin. North Am. 29:381–393. 19389588 10.1016/j.iac.2009.02.011

[45] Pedersen, B. K. , and L. Hoffman‐Goetz . 2000 Exercise and the immune system: regulation, integration, and adaptation. Physiol. Rev. 80:1055–1081. 10893431 10.1152/physrev.2000.80.3.1055

[46] Mackinnon, L. T. 2000 Chronic exercise training effects on immune function. Med. Sci. Sports Exerc. 32:S369–S376. 10910293 10.1097/00005768-200007001-00001

[47] Goldacre, M. J. , C. J. Wotton , and D. G. Yeates . 2009 Associations between infectious mononucleosis and cancer: record‐linkage studies. Epidemiol. Infect. 137:672–680. 18840316 10.1017/S0950268808001246

